# MR-Linac–Based SBRT for Prostate Cancer: Dosimetric Benefits for Urethral Sparing Compared to VMAT and Tomotherapy

**DOI:** 10.3390/cancers18040568

**Published:** 2026-02-09

**Authors:** Eva Y. W. Cheung, Darren M. C. Poon, Gavin C. K. Chan, Renee W. S. Ma, Jessie S. Y. Wong, Y. Nip, Connie N. K. Lam, K. P. Fong

**Affiliations:** 1 Department of Diagnostic Radiology, LKS Faculty of Medicine, University of Hong Kong, Hong Kong; 2 Department of Radiotherapy, Hong Kong Sanatorium and Hospital, Hong Kong; 3 School of Medical and Health Sciences, Tung Wah College, Hong Kong

**Keywords:** prostate cancer, stereotactic body radiotherapy, MR-Linac, VMAT, urethra sparing

## Abstract

**Background**: Stereotactic body radiotherapy (SBRT) for prostate cancer delivers high doses in few fractions but poses challenges in sparing adjacent organs at risk (OARs), particularly the intra-prostate urethra, bladder, rectum and penile bulb. Magnetic resonance-guided radiotherapy (MRgRT) using MR-Linac offers superior soft-tissue visualization and daily adaptive planning, potentially reducing OAR dose while maintaining target coverage. This study aimed to compare dose–volume parameters among MR-Linac (ML), volumetric modulated arc therapy (VMAT), and Tomotherapy (HT) plans for prostate SBRT. **Methods**: Thirty patients with localized prostate cancer were retrospectively analyzed. For each patient, three plans were generated: ML, VMAT and HT, using identical prescription and planning objectives. Dose–volume histogram (DVH) metrics were evaluated for clinical target volume (CTV), planning target volume (PTV), and OARs. Statistical comparisons were performed using non-parametric Friedman’s Test with post hoc Bonferroni test, with significance set at a *p* < 0.05. **Results**: CTV coverage was comparable across all modalities. ML achieved significantly higher PTV Dmin and near-maximum doses compared to VMAT and HT. Notably, ML provided substantial urethral sparing, reducing Dmax and Dmean by approximately 3.3 Gy compared to both VMAT and HT (*p* < 0.001). Rectal dose metrics were also lower with ML, while bladder and penile bulb doses showed minor increases (<3.5 Gy), considered clinically negligible. Femoral head doses were reduced in ML plans. **Conclusions**: MR-Linac planning for prostate SBRT offers meaningful dosimetric advantages, particularly in intra-prostate urethra urethral dose reduction, without compromising target coverage. These findings support incorporating MR-guided adaptive workflows into SBRT protocols to enhance OAR protection and potentially reduce treatment-related toxicity.

## Simple Summary

Prostate cancer is commonly treated with stereotactic body radiotherapy (SBRT), which delivers high radiation doses in a few sessions. While effective, SBRT poses challenges in protecting nearby organs at risk (OARs), such as the urethra, bladder, and rectum, from excessive radiation exposure. This study compared three planning techniques—MRI-guided radiotherapy using MR-Linac, volumetric modulated arc therapy (VMAT), and Tomotherapy—across 30 patients to evaluate differences in target coverage and OAR dose. All plans achieved comparable coverage of the prostate; however, MR-Linac demonstrated a significant reduction in urethral dose, lowering both maximum and mean doses by approximately 3.3 Gy compared to VMAT and Tomotherapy. Rectal and femoral head doses were also reduced with MR-Linac, while minor increases in bladder and penile bulb doses were observed but remained clinically acceptable. These findings highlight the potential of MR-Linac to improve treatment precision and reduce toxicity risk, supporting its integration into prostate SBRT protocols.

## 1. Introduction

Prostate cancer remains one of the most prevalent malignancies in men worldwide, and external-beam radiotherapy is a cornerstone of management for localized disease across risk groups. Over the past decade, stereotactic body radiotherapy (SBRT) has gained traction based on its radiobiologic rationale (low tumor α/β) and convenience, with multi-center randomized and prospective data supporting oncologic efficacy comparable to longer regimens and acceptable toxicity when delivered with contemporary techniques and image guidance [1,2,3]. Nonetheless, the close apposition of critical genitourinary (GU) and gastrointestinal (GI) organs at risk (OARs), particularly the intra-prostate urethra, bladder, rectum, and penile bulb, render dose escalation and margin reduction challenging with conventional CT-guided workflows such as VMAT or helical Tomotherapy, which rely on cone-beam CT (CBCT) and surrogates like fiducials for target verification and motion management [1,4]. These limitations motivate exploration of MR-guided platforms that provide superior soft-tissue contrast, facilitate daily adaptive planning, and enable real-time motion monitoring to improve dosimetric precision and reduce normal-tissue burden.

Magnetic resonance–guided radiotherapy (MRgRT) using integrated MRI linear accelerators (MR-Linac) represents a paradigm shift for prostate SBRT. The technology permits on-couch acquisition of high-contrast MR images and daily plan adaptation (“adapt-to-shape” or “adapt-to-position”), while cine MRI enables continuous intrafraction monitoring and automatic treatment interruption when motion exceeds tolerance [5]. Critically, MRgRT has been shown to support clinically safe reductions in CTV-to-PTV margins of ~2–3 mm—an intervention that directly curtails OAR dose exposures without compromising coverage [6]. Motion studies and early clinical series indicate that prostate motion remains within 3 mm for the majority of beam-on time on a 1.5 T MR-Linac, substantiating margin reduction in the absence of gating, and prospective MRgRT cohorts report feasibility and favorable short-term toxicity profiles in ultra hypo-fractionated regimens [7,8,9].

Beyond toxicity reduction, MR-Linac platforms may enable biologically guided intensification in selected settings, including focal boosting to dominant intraprostatic lesions (DILs) identified on multiparametric MRI and/or PSMA PET [10]. Early phase studies of PET/MRI-directed simultaneous in-field boost have demonstrated acceptable early toxicity and promising dosimetric control, while randomized phase II work is testing MR-guided two-fraction SBRT vs. five fractions, reflecting confidence in motion management and margin control afforded by MR guidance [11]. In parallel, analyses linking intraprostatic urethral dose to urinary toxicity across prospective SBRT trials underscore the clinical importance of urethral sparing; MR-Linac’s direct visualization of the urethra and continuous motion monitoring create a technical pathway to reduce urethral Dmax and Dmean while maintaining target coverage [12].

To address this gap, we conducted a dosimetric comparison in 30 men with localized prostate cancer, generating VMAT, Tomotherapy (HT), and MR-Linac plans per patient under standardized constraints. We hypothesized that MR-Linac planning would achieve comparable or superior CTV/PTV coverage while reducing the dose to critical OARs—most notably the intra-prostatic urethra relative to VMAT and HT. This study aimed to quantify differences in coverage metrics (Dmin, D95, near-maximums) and OAR dose–volume parameters (urethra, bladder, rectum, penile bulb, femoral heads), and to contextualize these findings against emerging randomized MR-guided data to inform protocol development for prostate SBRT.

## 2. Materials and Methods

### 2.1. Patient Selection

Thirty patients with primary prostate cancer who had received radical radiotherapy in the clinical oncology department of Hong Kong Sanatorium and Hospital (HKSH), Hong Kong, from 2020 to 2021 were recruited retrospectively. Patients who were diagnosed with adenocarcinoma of prostate and had localized disease of stage T1 to T2cN0M0 at a low or intermediate risk (less than 15%) for nodal metastases as defined by the Roach formula [2/3 × Prostate Specific Antigen (PSA) + 10 × (Gleason Score -6) [13] were included in this study. Patients who were treated diagnosed with nodal or distant metastases; received surgical resection of prostate; with prior radiotherapy to prostate or pelvis region; or with either unilateral or bilateral metal hip prosthesis were excluded.

### 2.2. Radiotherapy Treatment Simulation and Immobilization

Simulator CT images were acquired from the SOMATOM Confidence™ RT Pro (Siemens Healthcare GmbH, Erlangen, Germany) at HKSH. The scan range was from L3 lumbar spine to 5 cm below the perineum, with the slice thickness of 2 mm. Patients were simulated in supine position with handgrip, kneeSTEP and feetSTEP as immobilization. An endorectal balloon (QLARD, Miami, FL, USA) filled with 50–90 mL normal saline was used to stabilize the rectal volume and to minimize the rectal volume to be irradiated [14]. Patient were simulated with full bladder (130–160 mL confirmed by ultrasound), in order to displace the bowel superiorly further away from the treatment field, and to minimize the bladder size variation between fractions so as to keep the prostate within the planned treatment position [15]. MRI simulation was acquired from a 1.5 T scanner with a three-dimensional T2-weighted turbo spin echo (3D-T2W-TSE) sequence, aligned with daily MR-LINAC imaging parameters.

### 2.3. Image Registration and Structure Contouring

Simulated CT images, MRI and radiotherapy structure sets of each patient were anonymized and collected from HKSH. All images were co-registered and were used to delineate the clinical target volume (CTV) and the planning target volume (PTV) by radiation oncologists of HKSH, with reference to the departmental protocol. The organs-at-risk (OARs) were delineated by radiation therapists and dosimetrists of HKSH, including the rectum, bladder, femoral heads, bowels, penile bulb, sacral nerve, testes, penis and caudal equina. The intra-prostate urethra was contoured by the investigators of this study, with references to the co-registered MR images. All the contours were approved by certified medical dosimetrist before the radiotherapy planning procedure.

### 2.4. Ethics Approval

The ethics application was submitted to and has been approved by the Hong Kong Sanatorium and Hospitals, HKSH medical Group Research Committee (HKSH RC No: 2022-15).

### 2.5. Treatment Design and Planning

Stereotactic body radiation therapy (SBRT) was prescribed to all patients for this study. The dose prescription was 7.25 Gy per fraction (7.25 Gy/fr) at the PTV, 2–3 fractions per week for 5 fractions to a total of 36.25 Gy [16]. This dose regimen is recognized and recommended by National Comprehensive Cancer Network (NCCN) [17], the American Society for Radiation Oncology (ASTRO) [18] and the American Society of Clinical Oncology (ASCO) [19]. Three treatments plans were planned for each patient, including MRI-Linac (ML), volumetric modulated arc therapy (VMAT) and Helical tomotherapy (HT).

ML plans were planned using the Elekta Monaco^®^ Treatment Planning System v5.40 (Elekta, Stockholm, Sweden) with a Monte Carlo algorithm for Unity MRI-Linac. Photon beam energy (7 MV) was delivered with 6 gantry rotations per minute. An MLC-tracking system was employed with 11 fixed beams at gantry 0°, 30°, 60°, 80°, 100°, 120°,150°, 220°, 250°, 290°, and 320° was used for dose delivery. The Monte Carlo dose engine (GPUMCD) was utilized to account for magnetic field dose effect.

VMAT plans were planned using Eclipse Radiotherapy Treatment Planning System (Varian Medical Systems, Palo Alto, CA, USA), v15.6. with the progressive resolution optimizer (PRO3, v15.6, Varian Medical systems, Palo Alto, CA, USA). Photon beam energy (6 MV) had a maximum dose rate of 1000 monitor units (MU) per minute, and gantry speed was set at 4.8° per second. The field sizes were customized using a high-definition millennium multi-leaf collimator (HD-MMLC) 120 with a sliding window and were custom fitted in Eclipse Treatment Planning System with the arc geometry tool. Each plan consisted of 2 coplanar arcs: the first arc is a clockwise arc from gantry 181° to 179° with a fixed collimator angle at 30°, and the second arc is a counterclockwise arc from gantry angle 179° to 181° with a fixed collimator angle at 330. The collimator rotation was set at 30° and 330° for clockwise and anticlockwise arcs, respectively, to minimize the effect of inter-leaf leakage.

HT plans were planned using the Precision™ v3.3 (Accuray, Sunnyvale, CA, USA) for Accuray Radixact™. Photon beam energy (6 MV) and a 64-leaf binary MLC system were used [20]. All plans were optimized with Convolution Superposition algorithm. Dose calculation was performed with Monte Carlo algorithm using fine dose calculation grid with a resolution of 256 × 256 matrix in the xy-plane and 3 mm in the z-plane. Three parameters were input manually before optimization, including (1) the field width, which is the fan beam width in craniocaudal direction; (2) pitch which is defined as the ratio between the traveled distance of the couch per gantry rotation; (3) modulation factor, which governs the opening and closing speed of the MLCs. For each patient, a field width of 2.5 mm with dynamic jaw mode, pitch value of 0.287 and modulation factor of 2.4 were applied. In addition, the “Exit Only” and “Never” directional blocks were employed to minimize the radiation exposure to the rectum and the testes [21]. The maximum dose rate was set to 1180 monitor unit per minute (MU/min).

### 2.6. Treatment Plan Optimization

All three plans were optimized with reference to the same set of dose constraints in order to achieve plan acceptance. Also, identical constraints weighting was applied for each plan, this is to ensure the dose-volumetric parameters obtained were solely corresponded to the planning techniques but not related to the effects of weighting. Details of the dose constraints and weighting are listed in Table 1.

### 2.7. Inter-Planner Variability

To minimize inter-planner variability, all investigators completed 40 h of theoretical and hands-on practical training in treatment planning prior to study initiation. The MR-Linac (ML) plans were generated by clinical staff, while other investigators were responsible for generating VMAT, and Tomotherapy (HT) plans for every patient. Planning procedures were standardized across modalities. Before commencing the study, all planners performed exercises on four sample planning CT datasets, and their performance was evaluated using dose–volume metrics. Planners were permitted to begin study planning only after meeting the specified planning objectives for each technique [22,23]. All plans were evaluated and approved by certified medical dosimetrist before data collection.

### 2.8. Treatment Plan Evaluation

Two certified medical dosimetrists checked and approved all the plans. The following criteria were employed as planning goal for each plan:

1. More than 95% of CTV and PTV should receive 100% of prescribed dose, i.e., 36.25 Gy and 32.5 Gy respectively.
2. Less than 0.03 cc of CTV and PTV should receive less than 38.78 Gy (107% of the prescribed dose of CTV)
3. The maximum point dose should be confined in PTV, and should be less than 43.5 Gy (120% of the prescribed dose of CTV)
4. All dose to OAR should meet the dosimetric criteria listed in Table 1.

For plan quality evaluation, the homogeneity index and the conformity index were calculated as following,

Homogeneity index was calculated as:

(1) HI = (D1% − D99%)/DP

where D1% is the dose received by 1% (volume) of planning target volume (PTV), D99% is the dose received by 99% of PTV and DP is the prescribed dose (36.25 Gy) [24]. The D1% and D99% were used to account for the sharp dose falloff in SBRT plans.

Conformity Index was calculated as:

(2) $$Conformity\;Index\;(CI)=\frac{V_{Tref}}{V_{T}}\times \frac{V_{Tref}}{V_{ref}}$$

where *V_Tref_* is the volume of CTV receiving the prescribed dose (36.25 Gy), *V_T_* is the total volume of CTV and *V_ref_* is the volume receiving the prescribed dose (36.25 Gy). The *CI* ranged from 0 to 1, where a value closer to 1 indicated the dose coverage of target volume conformed more to the PTV [25].

Dose–volume evaluation

Dose–volume histograms (DVH) were generated for each plan, i.e., ML, VMAT and HT plan for each patient. All plans were evaluated qualitatively and quantitatively using standardized dose–volume (DV) parameters in view of dose to target volumes, dose homogeneity, dose conformity, and dose to OAR. The parameters used for the evaluation of CTV and OAR structures were listed in Table 2.

### 2.9. Statistical Analysis

IBM Statistical Product and Service Solutions (SPSS) (v25.0) was employed to perform the statistical analysis. A normality test was performed to confirm the normality of data distribution. If data are not normally distributed, a non-parametric Friedman test is used to detect difference in DV parameters among the three SBRT plans. A *p*-value ≤ 0.05 would be considered statistically significant in statistical tests. A post hoc test using Wilcoxon signed-rank test for individual pairwise comparisons with Bonferroni correction to control for Type I errors.

## 3. Results

### 3.1. Patient Demographic

Between June 2020 to December 2021, a total of 30 patients with PSMA-PET diagnosed prostate cancer were recruited into this study. CT images, MRI images, RT structure set were collected. The mean age 69.47 ± 8.14 (ranged 51–83) years old, with mean CTV of 51.7 cm^3^ ± 28.84 cm^3^.

### 3.2. Plan Evaluation—CTV DV Parameters Compliance

The overall dose–volume (DV) parameters for the clinical target volume (CTV) were comparable among MR-Linac (ML), VMAT, and HT plans. Statistically significant reductions in Dmax, Dmean, D1cc, D50%, D2%, and D1% were observed for ML compared to VMAT, with similar findings when comparing ML to HT. However, the absolute dose differences ranged from 0.07 to 0.21 Gy, indicating a minimal clinical impact. Detailed results are presented in Table 3.

### 3.3. Plan Evaluation—Dose Constraints Compliance

For plan acceptance, a predefined set of dose constraints was required. Regarding CTV and PTV coverage, it is noteworthy that the VMAT plan did not meet the PTV Dmin criterion, achieving only 29.71 Gy, which represents an underdose of approximately 1.2 Gy. When comparing MR-Linac (ML) to VMAT, statistically significant higher doses were observed for CTV D_0.03 cc (increase of 2.188 Gy), PTV Dmin (increase of 2.209 Gy), and PTV D_0.03 cc (increase of 7.043 Gy). Similar trends were noted when comparing ML to HT, with higher doses for CTV D_0.03 cc (increase of 2.42 Gy) and PTV D_0.03 cc (increase of 6.761 Gy.

Regarding organ-at-risk (OAR) dose constraints, the MR-Linac (ML) plan demonstrated significantly lower rectal exposure compared to VMAT, with reductions in the volume receiving 34.4 Gy (by 0.377 cc) and 18.125 Gy (by 1.569%). Additionally, significant decreases in maximum dose (Dmax) to the left and right femoral heads were observed, by 2.073 Gy and 2.369 Gy, respectively. However, the ML plan showed a higher percentage of bladder volume receiving 18.125 Gy, with an increase of 3.21% compared to VMAT, and the penile bulb exhibited a higher Dmax by 3.62 Gy. Similar trends were noted when comparing ML to HT, with the rectal volume receiving 34.4 Gy increasing by 0.04 cc and the penile bulb Dmax increasing by 1.203 Gy. Details are listed in Table 4.

### 3.4. Additional Dose–Volume (DV) Parameters Were Assessed for Gastrointestinal (GI) and Genitourinary (GU) OARs

For the rectum, beyond the previously reported volumes receiving 18.125 Gy and 34.4 Gy, the dose to 2% of rectal volume was 0.53 Gy lower in the MR-Linac (ML) plan compared to VMAT. In contrast, when comparing ML to HT, the rectal volume receiving 24 Gy was 0.86% higher, and the mean dose increased by 0.73 Gy.

For the bladder, ML showed a 0.92% more volume receiving 29 Gy and a 0.52 Gy increase in mean dose, although the maximum dose was 0.38 Gy lower. Similar trends were observed in the ML versus HT comparison, with a 0.06% increase in bladder volume receiving 29 Gy, a 0.07 Gy reduction in maximum dose, and a 0.09 Gy increase in the dose to 2% of bladder volume. These differences were minimal.

For the penile bulb, ML delivered significantly higher doses compared to VMAT, with increases of 3.62 Gy in Dmax, 2.75 Gy in Dmean, and 5.92 Gy for the dose to 2% of the volume. When comparing ML to HT, Dmax was 1.21 Gy higher, and the dose to 2% of the volume increased by 1.22 Gy.

**Figure 1:**
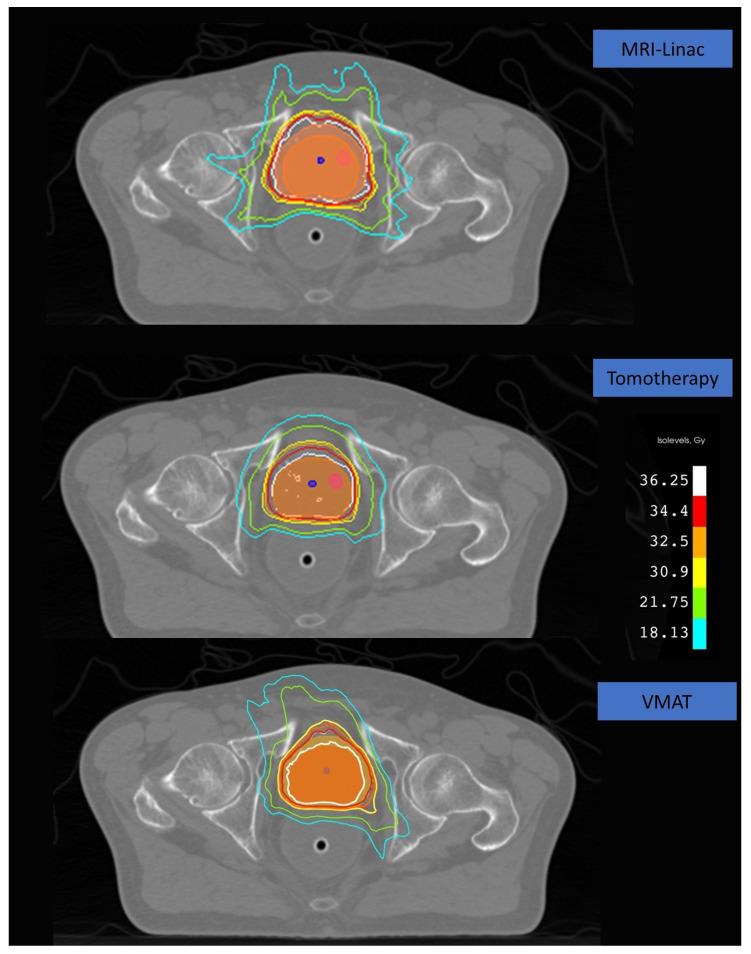
CTV is the red shaded region. PTV is the yellow shaded region. Urethra is the blue shaded region. The isodose lines from inner to outer represented the following isodoses: 36.25 Gy (white), 34.4 Gy (red), 32.5 Gy (orange), 30.9 Gy (yellow), 21.75 Gy (green), and 18.13 Gy (cyan).

For the intra-prostate urethra, ML achieved substantial dose reductions, with Dmax and Dmean decreased by 3.32 Gy and 3.26 Gy, respectively, resulting in mean values of 34.65 ± 0.17 Gy for Dmax and 32.40 ± 2.37 Gy for Dmean. A similar trend was observed in the ML versus HT comparison, with reductions of 3.08 Gy in Dmax and 3.17 Gy in Dmean. Details are listed in Table 5. The typical dose distribution of three plans for the same patient as shown in Figure 1.

## 4. Discussion

### 4.1. Clinical Target Volume (CTV): Coverage and Dosimetric Precision

Across the 30-patient cohort, the CTV DV parameters were broadly comparable between ML, VMAT and HT, with ML demonstrating statistically higher near-maximum dose metrics (D0.03 cc) relative to both VMAT and HT in our dataset. Although several absolute difference were modest, they are directionally consistent with the expected benefits of on-couch MRI visualization and daily adaption, which mitigate inter-and intrafraction anatomical changes (bladder filling, rectal distension, prostate drift) and support tighter margins without compromising target coverage [5,26]. Prior dosimetric studies have shown that when identical margins are used, ML plans quality is comparable to VMAT; however, MR guidance enables margin reduction, improving OAR sparing while preserving CTV coverage, which is an effect we recapitulate in the current comparison [27]. These observations align with evidence that MR-guided planning increases confidence in target volume delineation, and may translate into lower toxicity at equivalent tumor control in SBRT regimens [28,29].

### 4.2. Planning Target Volume (PTV) Minimum Dose and Potential Boost

A salient finding was the VMAT plan’s failure to meet the PTV Dmin acceptance criterion (underdose by 1.2 Gy), while ML achieved significantly higher PTV Dmin and near-maximum PTV D0.03cc. Clinically, raising Dmin reduces cold-spot risk and the likelihood of geographic miss, which is particularly consequential in SBRT where per-fraction dose is high [28]. The ML advantage is plausible given daily adapt-to-shape/position workflows, real-time motion monitoring, and treatment pausing when the prostate drifts out of tolerance, which are the capabilities that CT-based Linac has not provided [5,26]. Moreover, improved PTV coverage strengthens the foundation for biologically guided focal boost strategies to dominant intraprostatic lesions identifiable on MRI, a direction increasingly discussed for MR-guided SBRT [4,29]. These benefits must be weighed against operational trade-offs, that is longer treatment time and greater resource intensity for ML, though recent work demonstrates meaningful efficiency gains when VMAT delivery techniques are implemented on MR-Linac without compromising plan quality [8].

### 4.3. Organ at Risk: Dose Reduction and Clinical Interpretation

The ML plans reduced rectal exposure for key metrics, including lower volumes at 18.125 Gy and 34.4 Gy and lower dose to 2% of rectal volume, with only minimal increases in selected parameters versus HT. Lower rectal DVH burden is clinically relevant because it correlates with decreased acute/late GI toxicity (e.g., rectal hemorrhage), a signal echoed in comparative cohorts and randomized data for MR-guided versus CT-guided SBRT [4,29]. The consistency between our dosimetric findings and toxicity reductions in our previous study strengthens the argument that adaptive MR guidance yields tangible rectal sparing in practice [30].

ML plans showed small increases in bladder volume at intermediate dose (e.g., V29 Gy) and in mean dose, alongside a slight reduction in Dmax. The magnitudes were minimal and likely clinically modest, but they warrant routine monitoring given the relationship between bladder dose and urinary irritative/obstructive symptoms [29]. In MR-guided workflows, the trade-off may reflect margin tightening around the prostate while preserving target coverage. To prepare the bladder with reference to protocol and adaptive imaging when bladder filling exceeds thresholds can further attenuate bladder dose creep [8].

A key finding of this study is the significant reduction in intra-prostate urethral dose with MR-Linac (ML), with approximately 3.3 Gy lower Dmax and 3.3 Gy lower Dmean, achieved while maintaining adequate CTV and PTV coverage. The urethra is among the most dose-sensitive structures in prostate radiotherapy, and elevated urethral doses are strongly associated with stricture formation, urinary retention, and lower urinary tract symptoms, particularly in hypo-fractionated SBRT regimens [28,29]. In a meta-analysis of 23 clinical trials involving over 2000 SBRT patients, Leeman et al. demonstrated a clear association between intraprostatic urethral dose and treatment-related toxicity, with each 1 Gy increase corresponding to a 1% rise in late grade ≥2 toxicity and a 0.8% increase in acute grade ≥2 toxicity [12]. The combination of improved soft-tissue visualization for urethral delineation and daily adaptive planning in ML likely accounts for the observed dose reduction. Clinically, these urethral-sparing gains are highly relevant for mitigating toxicity and preserving quality of life, supporting the incorporation of explicit urethral constraints and adaptive triggers into MR-guided SBRT protocols [12,31].

ML plans exhibited modest increase in penile bulb Dmax and Dmean versus VMAT and HT. while the penile bulb is a surrogate for erectile structures, dose-function relationships are heterogenous, and small dose increments may be clinically tolerable, especially when balanced against urethral or rectal sparing [29]. Nonetheless, acknowledging this potential trade-off is prudent, and incorporating penile bulb dose optimizations into ML adaptive planning may help minimize any unintended dose increase [31].

ML plans reduced femoral head Dmax relative to VMAT. In current SBRT protocol, femoral head toxicity is uncommon, lower maximum dose further decreases any risk of osteoradionecrosis or fracture, particular in patient with pre-existing bone fragility [29].

### 4.4. Study Limitation

This study has several limitations. First, the analysis was based on a dosimetric comparison only, without correlating findings to clinical outcomes such as toxicity or quality of life measures. While dose reductions to critical structures like the urethra are promising, their actual impact on patient-reported symptoms and long-term complications remains to be validated in multi-institutional trials [12]. Second, the sample size of 30 patients, although adequate for detecting dosimetric differences, may limit generalizability across diverse anatomical variations and treatment settings. Larger, multi-institutional cohorts would strengthen external validity. Third, all plans were generated under idealized conditions using standardized constraints. The real-world workflow factors such as treatment time, adaptive decision making and patient compliance were not addressed. These operational aspects are critical for evaluating the practicality of MR-Linac adoption.

### 4.5. Study Novelty and Future Directions

This study leverages triple modality planning within the same cohort to highlight the urethral-sparing advantage and improved PTV Dmin with ML while maintaining CTV and PTV coverage. It is evidence that supports the unique value of daily adaptive, real-time MR guidance in prostate SBRT.

ML entails longer treatment sessions and greater staff demand. The dosimetric gains demonstrated in this study require a larger patient cohort to contextualize it against the operational constraints. Also, minor increases in bladder and penile bulb doses were observed and should be managed through protocol refinements in the adaptive rules or objective weighting in planning [31].

In future studies, correlation studies should be carried out to evaluate dosimetry with patient-reported outcomes, and clinical graded toxicity to quantify clinical benefit. Also, with improved motion management and margin control in ML plans, focal boost planning can be applied to boost MRI-visible intraprostatic lesions for deeper hypo-fractionation SBRT. To confirm the durability of oncological outcomes and generalizability of toxicity reductions, multi-institution trials or prospective study may be an option.

## 5. Conclusions

This study compares the dosimetric parameters of MR-Linac, VMAT and Tomotherapy plans for prostate SBRT. It demonstrates that MR-Linac can achieve comparable target coverage while offering distinct advantages in organ-at-risk sparing, particularly for the urethra. Significant reductions in urethral Dmax and Dmean with MR-Linac highlight its potential to mitigate urinary toxicity, which is a consideration in high-dose SBRT regimens. Although improvement in rectal and femoral head doses was observed, increases in bladder and penile bulb doses were minimal and likely clinically negligible. The ability of MR-Linac to deliver higher minimum and near-maximum PTV doses without compromising OARs constraints underscores its suitability for adaptive workflow and future strategies such as focal boosting. The findings support the integration of MR-guided adaptive radiotherapy into prostate SBRT protocol, while emphasizing the need for prospective studies to validate clinical benefits, assess long term toxicity and optimize workflow efficiency.

## Figures and Tables

**Table 1:** Dosimetric criteria and weighting of target volumes and organs-at-risk in all SBRT plans.

Target Volume	Planning Objectives	Weighting
CTV	D_95%_ ≥ 36.25 Gy V_38.78 Gy_ < 0.03 cc D_min_ > 34.4 Gy (Variation acceptable: D_0.03 cc_ ≥ 34.4 Gy)	100%
PTV	D_95%_ ≥ 32.5 Gy V_38.78 Gy_ < 0.03 cc D_min_ > 30.9 Gy (Variation acceptable: D_0.03 cc_ ≥ 30.9 Gy)	100%
**OARs**	**Dose–volume constraints**	**Weighting**
Rectum	V_38.06 Gy_ < 0.03 cc V_34.4 Gy_ < 3 cc V_36.25 Gy_ < 10% V_29 Gy_ < 20% V_18.125 Gy_ < 50%	80%
Bladder	V_38.06 Gy_ < 0.03 cc V_32.625 Gy_ < 10% V_18.125 Gy_ < 50%	80%
Femoral heads	D_max_ < 30 Gy V_20 Gy_ < 10 cc (sum of both sides)	80%
Penile bulb	D_max_ < 36.25 Gy V_20 Gy_ ≤ 3 cc	50%

**Table 2:** Dose-volumetric parameters for plan evaluation.

Target Volumes	Dose-Volumetric Parameters
PTV, CTV	Volume receiving 95% of target dose (V_95%_), Maximum dose (D_max_), Minimum dose (D_min_), Mean dose (D_mean_), 99% (D_99%_), 98% (D_98%_), 95% (D_95%_), 50% (D_50%_), 2% (D_2%_), Homogeneity index, Conformity index
**OARs**	**Dose-volumetric parameters**
Rectum	V_18.125 Gy (50%)_, V_24 Gy_, V_29 Gy (80%)_, V_34.4 Gy (95%)_, V_36.25 Gy (100%)_, D_max_, D_mean_, D_2%_
Bladder	V_18.125 Gy_, V_29 Gy_, V_36.2 5 Gy_, D_max_, D_mean_, D_2%_
Left femoral head	V_20 Gy_, D_max_, D_2%_
Right femoral head	V_20 Gy_, D_max_, D_2%_
Intra-prostate Urethra	V_38.78 Gy_, D_0.03 cc_, D_max_, D_mean_
Penile bulb	V_29.5 Gy_, D_max_, D_mean_, D_2%_

**Table 3:** Dose-volumetric parameters of the CTV.

CTV DV Parameters	ML	VMAT	HT	ML − VMAT	*p*-Value	ML − HT	*p*-Value
	Mean ± S.D.	Mean ± S.D.	Mean ± S.D.	Difference		Difference	
**V_95%_ (%)**	99.77 ± 1.25	100.00 ± 0.002	100.00 ± 0.00	−0.23	>0.05	−0.23	>0.05
**Maximum Dose (Gy)**	38.36 ± 0.21	38.51 ± 0.26	38.47 ± 0.21	−0.15 *	0.00	−0.11 *	0.00
**Minimum Dose (Gy)**	34.74 ± 3.35	35.34 ± 0.43	35.26 ± 0.33	−0.60	>0.05	−0.52	>0.05
**Mean Dose (Gy)**	37.02 ± 0.15	37.12 ± 0.10	37.03 ± 0.08	−0.10 *	0.00	−0.01	0.28
**D_1cc_ (Gy)**	37.74 ± 0.14	37.80 ± 0.17	37.80 ± 0.13	−0.06 *	0.02	−0.07 *	0.00
**D_99%_ (Gy)**	35.95 ± 1.69	36.33 ± 0.12	36.16 ± 0.09	−0.38	0.91	−0.21 *	0.00
**D_98%_ (Gy)**	36.12 ± 1.33	36.42 ± 0.11	36.28 ± 0.07	−0.30	0.16	−0.16 *	0.00
**D_95%_ (Gy)**	36.38 ± 0.68	36.55 ± 0.11	36.43 ± 0.06	−0.16	0.36	−0.05 *	0.00
**D_50%_ (Gy)**	37.03 ± 0.13	37.12 ± 0.11	37.02 ± 0.08	−0.09 *	0.00	0.01	0.21
**D_2%_ (Gy)**	37.74 ± 0.13	37.82 ± 0.18	37.81 ± 0.13	−0.08 *	0.01	−0.07 *	0.00
**D_1%_ (Gy)**	37.86 ± 0.13	37.93 ± 0.25	37.94 ± 0.14	−0.07 *	0.04	−0.08 *	0.00
**Homogeneity** **Index**	0.0526 ± 0.0465	0.0442 ± 0.008	0.0489 ± 0.005	0.0084	1.00	0.0037	0.0001
**Conformity Index**	0.7684 ± 0.0861	0.7880 ± 0.08	0.7734 ± 0.05	−0.0195	0.91	−0.0050	0.0037

* indicates *p* < 0.05, representing a statistically significant difference.

**Table 4:** Dose constraints compliance.

Structures	Dose Constraints	ML	VMAT	HT	ML—VMAT	*p*-Value	ML—HT	*p*-Value
		Mean ± S.D.	Mean ± S.D.	Mean ± S.D.	Difference		Difference	
**CTV**	D95% ≥ 36.25 Gy	36.44 ± 0.75	36.55 ± 0.11	36.49 ± 0.06	−0.106	0.117	−0.054	1.000
	V38.78 Gy < 0.03 cc	0.00 ± 0.001	0.00 ± 0.001	0.00 ± 0.004	0.000	>0.05	−0.001	>0.05
	Dmin > 34.4 Gy	34.84 ± 3.42	35.34 ± 0.43	35.26 ± 0.33	−0.496	>0.05	−0.412	>0.05
	D0.03 cc ≥ 34.4 Gy	38.20 ± 0.17	36.02 ± 0.44	35.78 ± 0.24	2.188 *	0.000	2.420 *	0.000
**PTV**	D95% ≥ 32.5 Gy	34.77 ± 1.41	34.29 ± 0.39	34.13 ± 0.50	0.485	0.974	0.637	0.345
	V38.78 Gy < 0.03 cc	0.00 ± 0.001	0.00 ± 0.001	0.00 ± 0.004	4.220	>0.05	4.220	>0.05
	Dmin > 30.9 Gy	31.92 ± 1.94	29.71 ± 0.96	31.09 ± 0.29	2.209 *	0.000	0.832	1.000
	D0.03 cc ≥ 30.9 Gy	38.74 ± 1.45	31.70 ± 0.48	31.98 ± 0.83	7.043 *	0.000	6.761 *	0.000
**Rectum**	V38.06 Gy < 0.03 cc	0.00 ± 0.00	0.00 ± 0.00	0.00 ± 0.004	0.000	>0.05	−0.001	>0.05
	V34.4 Gy < 3 cc	1.27 ± 0.99	1.65 ± 0.86	1.23 ± 0.95	−0.37 *	0.000	0.039 *	0.029
	V36.25 Gy < 10%	0.12 ± 0.15	0.13 ± 0.16	0.12 ± 0.17	−0.006	>0.05	0.004	>0.05
	V29 Gy < 20%	4.04 ± 1.46	4.24 ± 1.28	3.88 ± 1.25	−0.190	>0.05	0.173	>0.05
	V18.125 Gy < 50%	13.15 ± 3.33	14.72 ± 3.84	12.52 ± 2.87	−1.550 *	0.085	0.636	0.467
**Bladder**	V38.06 Gy ≤ 0.03 cc	0.07 ± 0.41	0.00 ± 0.001	0.00 ± 0.007	0.075	>0.05	0.074	>0.05
	V32.625 Gy ≤ 10%	4.59 ± 2.85	4.40 ± 2.85	4.51 ± 2.93	0.201	1.000	0.083	0.060
	V18.125 Gy ≤ 50%	22.65 ± 10.80	19.42 ± 10.82	22.04 ± 10.80	3.21 *	0.000	0.613	1.000
**Left Femoral Head**	Dmax < 30 Gy	13.06 ± 2.14	15.14 ± 3.30	12.83 ± 1.92	−2.073 *	0.009	0.235	0.467
	V20 Gy < 10 cc	0.0000 ± 0.00	0.06 ± 0.31	0.00 ± 0.00	−0.057	>0.05	0.000	>0.05
**Right Femoral Head**	Dmax < 30 Gy	13.1243 ± 2.16	15.49 ± 2.95	12.91 ± 2.02	−2.369 *	0.001	0.218	0.467
	V20 Gy < 10 cc	0.02 ± 0.13	0.03 ± 0.13	0.00 ± 0.00	−0.002	>0.05	0.024	>0.05
**Femoral Heads**	V20 Gy < 10 cc	0.40 ± 0.32	0.08 ± 0.34	0.00 ± 0.00	0.320	>0.05	0.402	>0.05
**Penile Bulb**	Dmax < 36.25 Gy	15.93 ± 11.66	12.31 ± 11.16	14.73 ± 11.32	3.620 *	0.000	1.206 *	0.000
	V20 Gy ≤ 3 cc	0.27 ± 0.62	0.11 ± 0.42	0.30 ± 0.66	0.166	0.467	−0.023	0.660

* indicates *p* < 0.05, representing a statistically significant difference.

**Table 5:** Dose-volumetric parameters of GI/GU OARs.

OAR DV Parameters		ML	VMAT	HT	ML—VMAT	*p*-Value	ML—HT	*p*-Value
		Mean ± S.D.	Mean ± S.D.	Mean ± S.D.	Difference		Difference	
**Rectum**	V_18.125Gy_ (%)	13.17 ± 3.10	14.72 ± 3.84	12.52 ± 2.87	−1.55 *	0.08	0.66	0.47
	V_24Gy_ (%)	7.8 8± 2.64	7.66 ± 2.05	7.03 ± 1.87	0.22	1.00	0.86 *	0.01
	V_29Gy_ (%)	4.02 ± 1.30	4.24 ± 1.28	3.88 ± 1.25	−0.22	>0.05	0.14	>0.05
	V_34.4Gy_ (cc)	1.2 8± 0.99	1.65 ± 0.86	1.23 ± 0.95	−0.37 *	0.00	0.04	0.12
	V_36.25Gy_ (%)	0.18 ± 0.32	0.13 ± 0.16	0.12 ± 0.17	0.05	>0.05	0.06	>0.05
	D_max_ (Gy)	36.88 ± 0.80	36.98 ± 0.57	36.92 ± 0.83	−0.10	>0.05	−0.05	>0.05
	D_mean_ (Gy)	10.34 ± 5.43	10.17 ± 1.43	9.62 ± 1.15	0.17	1.00	0.73 *	0.01
	D_2%_ (Gy)	32.02 ± 1.63	32.55 ± 1.45	31.95 ± 1.57	−0.53 *	0.01	0.07	0.36
**Bladder**	V_18.125Gy_ (%)	22.63 ± 10.51	19.42 ± 10.82	22.04 ± 10.80	3.21 *	0.00	0.58	1.00
	V_29Gy_ (%)	8.09 ± 4.15	7.17 ± 4.39	8.03 ± 4.26	0.92 *	0.00	0.06 *	0.01
	V_36.25Gy_ (%)	1.28 ± 1.40	0.90 ± 1.17	1.09 ± 1.14	0.38	0.32	0.20	0.32
	D_max_ (Gy)	37.13 ± 0.62	37.52 ± 0.73	37.20 ± 0.60	−0.38 *	0.00	−0.07 *	0.00
	D_mean_ (Gy)	9.27 ± 4.12	8.75 ± 3.85	10.03 ± 3.81	0.52 *	0.00	−0.76	1.00
	D_2%_ (Gy)	34.45 ± 2.09	33.96 ± 3.06	34.36 ± 2.23	0.49	0.91	0.09 *	0.00
**Penile**	V_29.5Gy_ (%)	4.85 ± 18.53	1.11 ± 4.32	1.56 ± 5.97	3.75	>0.05	3.29	>0.05
**Bulb**	D_max_ (Gy)	15.93 ± 11.66	12.31 ± 11.16	14.73 ± 11.32	3.62 *	0.00	1.21 *	0.00
	D_mean_ (Gy)	6.38 ± 7.41	3.63 ± 3.61	5.55 ± 4.85	2.75 *	0.00	0.83	1.00
	D_2%_ (Gy)	15.56 ± 12.00	9.64 ± 9.52	14.34 ± 11.23	5.92 *	0.00	1.22 *	0.01
**Intra-prostate** **urethra**	V_38.78Gy_ (cc)	0.00 ± 0.00	0.00 ± 0.00	0.00 ± 0.00	0.00	>0.05	0.00	>0.05
	D_0.03cc_ (Gy)	36.25 ± 6.85	37.62 ± 0.29	37.00 ± 1.10	−1.36	0.12	−0.74 *	0.04
	D_max_ (Gy)	34.65 ± 0.17	37.97 ± 0.34	37.74 ± 0.21	−3.32 *	0.00	−3.08 *	0.00
	D_mean_ (Gy)	32.40 ± 2.37	35.14 ± 2.70	35.23 ± 2.55	−3.26 *	0.04	−3.17 *	0.04

* indicates *p* < 0.05, representing a statistically significant difference.

## Data Availability

The data presented in this study are available on request from the corresponding author. The data are not publicly available due to privacy or ethical restrictions.
